# Primitive genotypic characteristics in umbilical cord neutrophils identified by single-cell transcriptome profiling and functional prediction

**DOI:** 10.3389/fimmu.2022.970909

**Published:** 2022-08-29

**Authors:** Yi Chen, Jiamin Huang, Zaiwen Guo, Zhechen Zhu, Yiming Shao, Linbin Li, Yunxi Yang, Yanzhen Yu, Lu Liu, Bingwei Sun

**Affiliations:** ^1^ Department of Burns and Plastic Surgery, Affiliated Suzhou Hospital of Nanjing Medical University, Suzhou, China; ^2^ Department of Burns and Plastic Surgery, Affiliated Huaian No.1 People’s Hospital, Nanjing Medical University, Huai’an, China

**Keywords:** umbilical cord blood, neonate, neutrophils, single cell, transcriptome

## Abstract

The function and heterogeneity of neutrophils in neonatal umbilical cord blood (UCB) have not been characterized. In this study, we analyzed the neutrophils in UCB and healthy adults using single-cell RNA sequencing analysis for the first time. We found that neutrophils divided into six subpopulations (G2, G3, G4, G5a, G5b, and G5c) with different marker genes and different functions under homeostasis. Compared with healthy adults, neutrophils of UCB were more naïve and have more obvious degranulation and activation functions. Moreover, we found significant differences in the amount and function of G5b cells between healthy adults and UCB. The amount of G5b group in UCB was lower, but it has more degranulation, secretion and activation functions. In addition, we noted a new subset of G5c labeled by CD52, which almost did not exist in UCB. Besides, its differential genes were enriched in terms such as protein synthesis and mRNA transcription. Furthermore, uncharacteristic transcription factors ZNF-276, ZNF-319 and ZNF-354A were identified in our study. In summary, we first examined the heterogeneity and functional diversity of neutrophils in UCB, and these data provided new insights into the mechanism of neutrophil-mediated diseases of neonates and the wider use of neutrophils in UCB.

## Background

The neutrophil is a major component of the innate immune system and are the first cells recruited to injured or infected sites. In the presence of abnormal neutrophil numbers or quality, it might disrupt the immune system’s homeostasis environment and contribute to the development or progression of disease. Neutropenia or neutrophil dysfunction would put patients at high risk for fatal infection (1). With the in-depth study of neutrophils, researchers have proved that neutrophil dysfunction developed many diseases, such as malignant tumors, autoimmune diseases and severe infections (2, 3). Granulocyte transfusion as one of the main treatment methods has a long history in treating patients with neutropenia or neutrophilic dysfunction, and can be used to prevent and treat infection (4). In addition, in recent years, with the emergence of immunotherapy, neutrophilic adoptive therapy also began to be used in the treatment of refractory infectious diseases and malignant tumors, and good therapeutic effect was achieved (5).

It was increasingly recognized that UCB, previously considered medical waste, was actually a valuable source of therapeutic cells (6). The cells of UCB were easier to collect than that of healthy adults, and their unique properties made them especially appropriate for cell therapy. The unique properties of these cells included their naive nature (7, 8), the high proportion of stem and progenitor cells (7, 9), and non-hematopoietic cells with therapeutic potential (8). But neutrophils in UCB, the most numerous of the immune cells, were rarely mentioned. There were no clear differences between UCB-derived neutrophils and healthy adults’ neutrophils in terms of how they function.

With the help of single-cell RNA sequencing(scRNA-seq), an important method for characterizing immune cells and their function, we preliminary explored the differences between neutrophils of UCB and adults. We provided the first reference map of neutrophils subsets in UCB, and the functional difference of neutrophils in UCB were explained. The results of our study lay the theoretical groundwork for the further study of UCB neutrophils.

## Result

To study the functional changes of neutrophils in UCB, we tested neutrophils with scRNA-seq in healthy adults and UCB. Neutrophils samples for sequencing were collected from peripheral blood of 25 healthy adults and UCB of 40 healthy neonates of full-term natural delivery (**Table 1**). Among them, 5 UCB samples and 5 peripheral blood samples from healthy adults were used for the detection of seRNA-seq. The magnetic bead separation was used for the separation of neutrophils.

**Table 1:** Clinical data of UCB (including maternity and neonate) and healthy adults submitted for examination.

Characteristics	Maternity	Neonate	Healthy adult
Number	n=40	n=40	n=25
Sex, female (%)	100%	45%	44%
Age (year)	26 (21-34)	N/A	25 (22-35)
Gestational age(week)	N/A	39 (38-41)	N/A
Delivery mode, spontaneous labor (%)	N/A	100%	N/A
Intrapartum fever (%)	0	N/A	N/A
GBS Colonization (%)	0	N/A	N/A
PIH (%)	0	N/A	N/A
Gestational diabetes (%)	0	N/A	N/A
Birth weight (g)	N/A	3447.29 ± 570.45	N/A
1min Apgar score	N/A	10	N/A
5min Apgar score	N/A	10	N/A
10min Apgar score	N/A	10	N/A
WBC count (10^9^/L)	7.49± 1.23	N/A	N/A
Neutrophil count (10^9^/L)	5.24 ± 1.01	N/A	N/A

Data presented as median (age range and birth weight); mean (standard deviation) or number (percentage); GBS, group B β hemolytic streptococcus; PIH, Pregnancy induced hypertension; WBC, white blood cell; N/A, not applicable.

### Conservative classification of neutrophils in UCB

**Figure 1:**
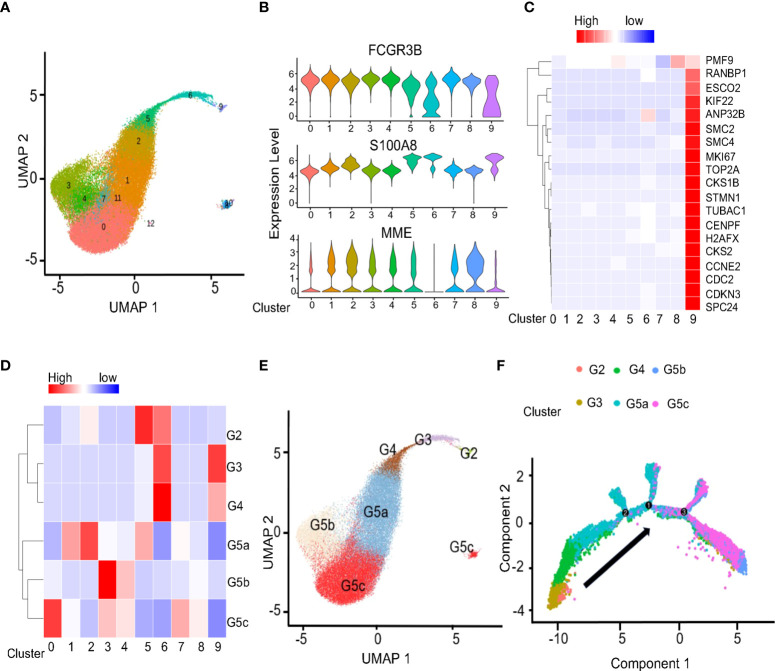
Conservative classification of neutrophils in UCB and healthy adults. **(A)** Uniform manifold approximation and projection (UMAP) of 45905 neutrophils from UCB and healthy adult. **(B)** Violin plots of the genes FCGR3B, S100A8 and MME in populations. **(C)** Heatmap showing row-scaled expression of cell cycle-related genes for 0-9 clusters neutrophils. **(D)** Correlation of scRNA-seq defined neutrophil clusters with the neutrophil subtypes reported by Xie et al. **(E)** Projection of new clustering results on UMAP. **(F)** Pseudo-time analysis of neutrophils. The order of cells was inferred from the expression of the most variable genes in all cells, and the direction of development was determined from biological features.

The 45905 high-quality cells were obtained through rigorous quality control (**Supplementary Figure 1A**). We identified 13 major cell populations by graph-based clustering (**Figure 1A**). With the known genetic markers (HBB, HBA1, and HBA2) of red blood cell (RBC) (10), groups 12 were considered RBC (**Supplementary Figure 1B**). Furthermore, 10 and 11 clusters were also characterized by a high percent of mitochondrial unique molecular identifier (UMI) count and low UMI count per cell (**Supplementary Figure 1C**). Therefore, in further analysis we discarded groups 10,11 and 12. FCGR3B and S100A8 are considered as specific marker genes of neutrophil (11), and CD10 encoded by MME is considered to be a surface marker of mature neutrophils (12). For these reasons, we identified groups 0-9 as neutrophils, and subgroups 6 and 9 as immature neutrophils (**Figure 1B**). The specific marker genes Kit and CD34 of granulocyte-monocyte progenitors (GMP) (13) are not expressed on neutrophils (**Supplementary Figure 1D**). As a result, no GMP was found in the neutrophils we collected. In addition, the correlation analysis based on cell subpopulation differential genes and cell cycle-related genes (**Figure 1C** and **Supplementary Table-Sheet 1**) suggested that 9 cluster could have a proliferative function in the neutrophil subsets. According to the study of Xie et al (14), neutrophils have been classified into eight subsets (G0-G5c) in bone marrow, tissue and circulation. Using the genetic markers present in the references (**Supplementary Table-Sheet 2**), we conducted correlation analysis between our data and the data set in the references (**Figure 1D**). At the same time, combined with the ScGeneModule analysis (**Supplementary Figure 1E**), the final classification of neutrophils was confirmed. The original cell subgroups were reclassified into G2-G5C (**Figure 1E**). As expected, a quasi-temporal analysis was performed on neutrophils, revealing that differentiation and maturation took place in a closely linked trajectory from G2 to G5c (**Figure 1F** and **Supplementary Figure 1F**).

### Characteristics of differentiation and development of neutrophil subgroups

**Figure 2:**
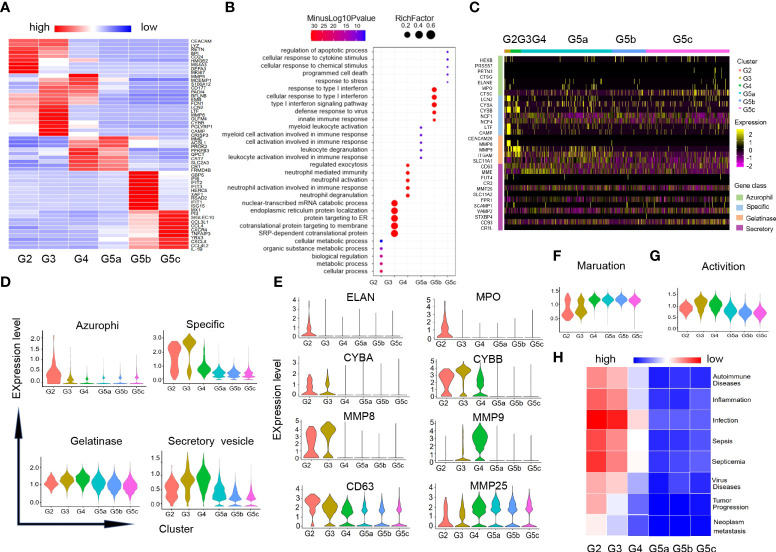
Characteristics of differentiation and development of neutrophils subgroups. **(A)** Heatmap of gene expression in each subgroup based on differentially expressed genes (DEGs). **(B)** Diagram of gene ontology (GO) about biological process (BP) analysis for genes within each subgroup. The following GO terms were shown with Benjamini-Hochberg corrected P-values <0.05 (one-sided Fisher’s exact test). **(C)** Heatmap illustrating four-level granule genes expression of neutrophils. **(D)** Violin diagrams of four-level granule genes function scores in each cluster. **(E)** Violin diagrams of representative genes related to four-level granules of neutrophils. **(F, G)** Violin plots of the functional scores of activation and maturation of neutrophil subsets. **(H)** Heatmap for correlation analysis between each subgroup and the disease.

Our data identified additional DEGs and characteristically expressed genes that distinguished each subgroup (**Figure 2A**). And these DEGs analysis also revealed that each subgroup of neutrophils was enriched for different GO-BP items (**Figure 2B**). In line with the differentiation and maturation process of neutrophils, neutrophils highly expressed MKI67, MS4A3 (**Figure 2A**) and other genes related to cell proliferation in G2 cells, and highly expressed aging marker CXCR4 in the more mature subset G5c. Apart from that, the genes (CXCL8, CCL4, IL-1B and TNFAIP3) coding chemokines and inflammatory factors were expressed at high level in G5c cells as well (**Figure 2A**).

There were four types of particles in neutrophils: azurophil granule, specific granule, gelatinase granule and secreted vesicle (15). Expression of granule genes was closely related to neutrophils differentiation and maturation. Scores of granule genes were calculated for each subpopulation based on gene expression level (**Supplementary Table-Sheet 3** and **Figures 2C, D**). Consistent with previous studies, the genes (ELANE, MPO and CD63) coding for the azurophil granule proteins were highly expressed in G2 cells. G3 and G4 cells had high expression of specific granule genes (CYBB, CYBA) and gelatinase granule genes (MMP8, MMP9). The related-gene MMP25 of secretory vesicle was up-regulated in the late stage of neutrophil differentiation (**Figure 2E**).

In addition, we measured the maturation and activation functional scores of each cluster based on the expression of related genes (**Supplement Table-Sheet 3**). As expected, group G5 was the most mature neutrophils (**Figure 2F**), while G3 and G4 cells showed the highest activation functional scores of all subsets (**Figure 2G**). To predict the association between each subgroup of neutrophils and diseases, we performed correlation analysis for each subgroup and diseases in published literatures based on the characteristic gene expression of each subgroup (**Figure 2H**). We found that G2-G3 populations can predict diseases including sepsis, inflammation, as well as tumor development and metastasis.

### Functional differences of neutrophils between UCB and adult

**Figure 3:**
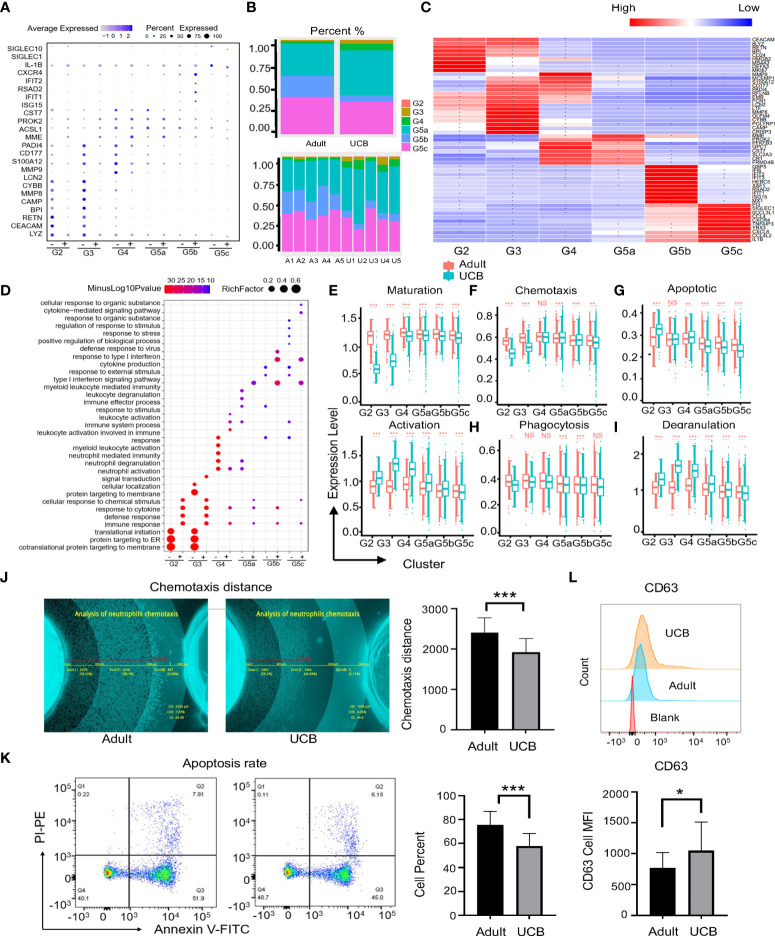
Functional differences among neutrophil subgroups between UCB and healthy adult. **(A)** Neutrophils conserved marker genes. ‘-’means neutrophils in UCB, and ‘+’ represents healthy adult neutrophils. **(B)** Plots of subpopulations in different groups and samples. **(C)** Heatmap of differentially expressed genes in subsets of neutrophils between healthy adult and UCB. Asters represent gene expression differences between UCB and healthy adults. **(D)** Scale diagram of GO-BP analysis of various subsets of neutrophils in healthy adults and UCB. ‘-’means DEGs up-regulated of adults, while ‘+’ represents DEGs up-regulated of UCB neutrophils. **(E–I)** Functional scores of neutrophil subgroups. Neutrophil maturation and activation **(E)**; Neutrophil chemotaxis **(F)** and apoptosis **(G)**; Neutrophil phagocytosis **(G)** and degranulation. **(J–L)** Neutrophil functional evaluation *in vitro*. Chemotaxis of neutrophils in healthy adults and UCB **(J)**. Neutrophil apoptosis in healthy adults and UCB **(K)**. The expression levels of CD63 in neutrophils of healthy adult and UCB **(L)**. Data represent means ± s.d. (n= 3–5) of two independent experiments. *p < 0.05, **p < 0.01, ***p < 0.001, ns, not statistically.

We analyzed genetic markers for neutrophils each subgroup in UCB and healthy adults, and neutrophils were divided into six cell subgroups (G2-G5C) in both groups (**Figure 3A**). However, there were significant differences among them. The main subgroups of healthy adults were G5a, G5b and G5c, while G5a and G5c were dominant in UCB (**Figure 3B**). In addition, to analyze functional differences of neutrophil subsets between adult and UCB, GO-BP analysis was performed according to DEGs (**Figure 3C**). There were significant differences in enrichment items among subsets (**Figure 3D**). The subsets of UCB are mainly enriched in degranulation, cell activation and secretion, while the subsets enriched in immune response and cytokine response of healthy adult.

Moreover, the maturation score of neutrophils in UCB was lower than that in healthy adults. In contrast, the neutrophils of UCB had higher activation scores in each subset (**Figure 3E**). The differences between the two groups were most significant in G2, G3 and G4 clusters. The functional scores of chemotaxes, phagocytosis and apoptosis of UCB neutrophil were down-regulated (**Figures 3F–H**). Interestingly, different from previous studies, the degranulation score of G2-G5C cells significantly increased in UCB (**Figure 3I**).

Next, we verified functions of UCB neutrophils *in vitro*. Consistent with the differences in gene expression, the chemotactic and apoptotic functions of UCB neutrophils were lower than those of healthy adults (**Figures 3J, K**), and the expression of degranulation related index CD63 was higher than that of healthy adults (**Figure 3L**). Besides, there was no significant difference in CD35 expression between the two groups (**Supplementary Figure 2A**), which was contributed to secretory vesicles. These results indicated that above functions may be regulated by related genes transcription. However, the phagocytic phenotype and genotype of neutrophils were inconsistent, there was no significant difference between adult and UCB (**Supplementary Figure 2B**). Data from these studies suggested that neutrophil phagocytosis might be driven by post-transcriptional or post-translational processing of related genes.

### Comprehensive analysis of the vulnerable subset of neutrophils in UCB—G5b subgroup

**Figure 4:**
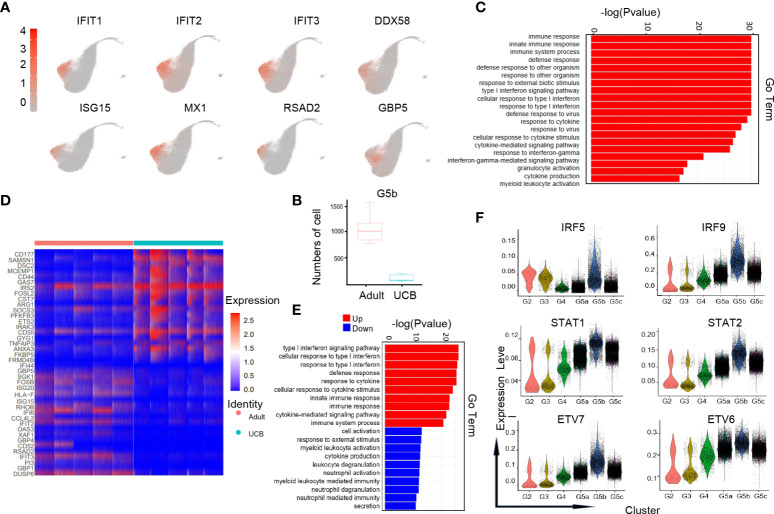
Characteristics of the G5b subgroup in UCB and adult. **(A)** UMAP of specific genetic markers in the G5b subgroup. **(B)** The amount of G5b cells in cord blood and healthy adults. **(C)** Go-BP analysis of differential genes in G5b subgroup compared with the other subgroups. GO terms with Benjamini-Hochberg-corrected P-values <0.05 (one-sided Fisher's exact test) are listed. **(D)** Heat maps of G5b genes differentially expressed in healthy adults and UCB. **(E)** GO-BP analysis of differential genes in healthy adults and UCB. Gene cluster entries in red represent differentially expressed genes up-regulated, while gene cluster entries in blue represent differentially expressed genes down-regulated. **(F)** Violin diagram of characteristic transcription factors in the G5b subgroup.

G5b cells with specific Interferon-stimulated gene (ISG) gene markers (**Figure 4A**) can predict diseases in the body. Previous studies have shown that the G5b subgroup could be rapidly amplified in the case of Escherichia coli infection, and play a role of sterilization and anti-infection (14). However, the amount of G5b subgroup was significantly reduced under early burn stress condition (16). Interestingly, our results showed that the amount of G5b cells in steady-state UCB was much lower than that in healthy adults (**Figure 4B**). Consistent with previous researches (14, 16), the functions of G5b compared with other clusters were mainly enriched in ISG related pathways, anti-infection and response to external stress, etc (**Figure 4C**).

To analyze the differences in G5b group function between UCB and healthy adults, we performed GO-BP analysis based on DEGs (**Figures 4D, E**). The results indicated that the G5b subpopulation in healthy adults were mainly enriched in immune response, ISG pathway and cytokine stimulation, while the G5b cells in UCB were mainly enriched in activation, degranulation and secretion of cells.

In addition, we also noticed that interferon-specific transcription factors IRF5 and IRF9 were highly expressed in the G5b subgroup (**Figure 4F**). Meanwhile, STAT1 and STAT2, as interferon signal sensors and transcriptional activators, were also highly expressed in G5b cells. Besides, ETV7 and its accessory gene ETV6 were up-regulated in G5b subset, which regulated cell development and differentiation.

### New subset of G5c (further classification)

**Figure 5:**
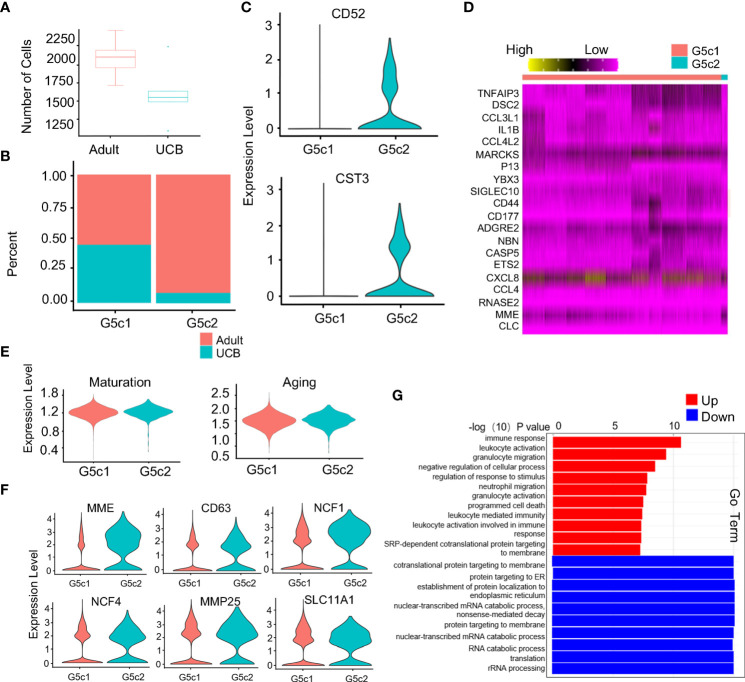
Specific expression of G5c subset in neutrophils. **(A)** Scale diagram of G5c in healthy adults and UCB. **(B)** Proportions of G5c1 and G5c2 in deferent group. **(C)** Violin diagram of G5c2 specific marker genes. **(D)** Heatmap of G5c1 and G5c2 differential genes based on DEGs. **(E)** Violin plots of the functional scores of activation and maturation of G5c1 and G5c2. **(F)** Violin diagrams of genes expression related with neutrophils four-level granules in G5c2. **(G)** GO-BP analysis based on DEGs between G5c1 and G5c2. Gene cluster entries in red represent differentially expressed genes up-regulated, while gene cluster entries in blue represent differentially expressed genes down-regulated.

Compared with healthy adults, the proportion of G5c cells in UCB was significantly reduced (**Figure 5A**). Interestingly, a specific subset of cells was present in the G5c population that was prevalent in the peripheral blood of healthy adults but almost absent in UCB (**Supplementary Figure 3A**). We speculated that this group of cells has a special function. Therefore, G5c was reclassified into G5c1 and G5c2 according to the characteristic genes (CD52, CST3) (**Figures 5B, C**). To clarify the function of this group of cells, G5c1 and G5c2 were re-analyzed for differential genes (**Figure 5D**). Based on DEGs analysis, we calculated neutrophil maturity and aging score, and found that there were no significant differences in maturity and aging score between the two groups (**Figure 5E**). As mentioned above, neutrophils granule biosynthesis is time-targeted. Therefore, we further analyzed the expression of granule genes in the two groups (**Supplementary Figure 3B**), and noticed that some genes (MME, CD63, MMP25, and NCF1) coding granule and secretion vesicle were up-regulated in G5c2 cells (**Figure 5F**). To clarify the functional differences between the two groups, GO-BP analysis was performed (**Figure 5G**). The results indicated that DEGs of G5c1 cells were mainly enriched in terms such as cell activation and immune response, however, G5c2 cells were enriched in functions like mRNA translation, protein synthesis and localization. At the same time, the DEGs of subsets were involved in the related pathways (**Supplementary Figures 3C, D**).

### Natural selection of UCB neutrophils for metabolic regulation

**Figure 6:**
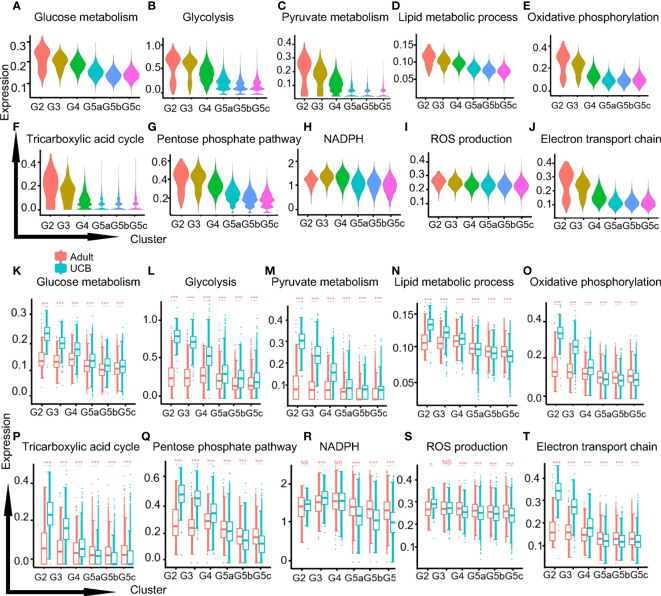
Metabolic characteristics of neutrophil subgroups. **(A–C)** The violin plots of glycol-metabolism-related functions scores in each subgroup. Glucose metabolism **(A)**, glycolysis **(B)** and pyruvate metabolism **(C)**. **(D)** The violin plot of lipid metabolism function score. **(E, F)** The violin plots of mitochondria related metabolism functional scores in each subset. Oxidative phosphorylation **(E)** and the tricarboxylic acid cycle **(F)**. **(G–J)** The violin plots of functions scores in metabolism-related ROS production of each subset. Pentose phosphate pathway **(G)**, NADPH **(H)**, ROS production **(I)**, electron transport chain **(J)**. **(K–T)** Box plots of function scores of the metabolic pathways in UCB and healthy adults. *p < 0.05, ***p < 0.001, NS, not statistically.

The metabolic activities of neutrophils were closely related to their differentiation, maturation and functional performance (17). Adenosine triphosphate (ATP) was produced by three main pathways: glycolysis, the tricarboxylic acid (TCA) cycle and oxidative phosphorylation (OXPHOS). Mitochondria mainly participates in TCA and OXPHOS metabolism. And mature neutrophils used ATP mainly provided by glycolysis. Our results showed that the functional scores of glucose-related metabolisms (glucose metabolism, glycolysis, and pyruvate) and lipid metabolism decreased progressively along with the differentiation and development of neutrophils (**Figures 6A–D, K–N**). This could be due to the fact that neutrophils need a large amount of ATP in the early process of proliferation and differentiation, and the cells were in a high catabolic state. One of the characteristics of the maturation of neutrophils was the decreased activity of their mitochondria and ATP production (18). It has also been confirmed in our study that TCA and OXPHOS functional scores of mitochondria metabolism-related were significantly up-regulated in G2-G4 cells and down-regulated in mature neutrophils (**Figures 6E, F, O, P**). Furthermore, the differences were more significant in UCB, which might attribute to the higher mitochondrial content and activity of neutrophils in UCB.

One of the products of pentose phosphate pathway (PPP) was nicotinamide adenine dinucleotide phosphate (NAPDH), and the production of reactive oxygen species (ROS) was mainly mediated by NOX produced by NADPH oxidase. The functional scores of PPP, NADPH and ROS production were relatively stable in healthy adult, while neutrophils in UCB were significantly up-regulated in G2-G3 cells and down-regulated in G5 cells (**Figures 6G–I, Q–S**). Besides, electron conduction chain mediated by mitochondrial also contributed to ROS production, and the functional score decreased in G5 cells (**Figures 6J, T**).

### Novel transcription factors of neutrophils in UCB

**Figure 7:**
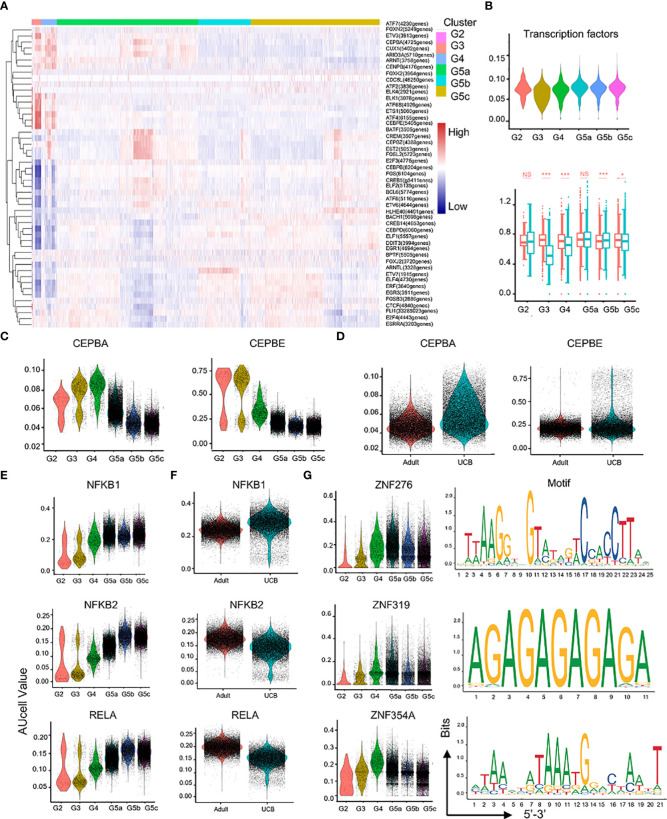
Characteristics of transcription and communication in neutrophil subsets. **(A)** Heatmap for transcription factors with different regulatory intensities in each subset of neutrophils. **(B)** Violin plot of characteristic transcription factors of neutrophils in each subset. Box plot of neutrophilic transcription factor scores between UCB and adults in each subset. **(C–F)** Violin plot of different activity of transcription factors in neutrophil subsets. **(G)** Activity and motif description based binding sites of three novel transcription factors in neutrophil subsets. *p < 0.05, ***p < 0.001, NS, not statistically.

In the process of neutrophil differentiation and development, different transcription factors are involved. To investigate the regulatory mechanisms of neutrophils in UCB during differentiation and maturation, we performed a SCENIC analysis (**Figure 7A**). Compared with healthy adults, transcriptional activity of UCB neutrophils in the G3 subset was significantly down-regulated (**Figure 7B**), suggesting potential functional changes in during G3 cells in UCB. Previous studies have confirmed that C/EBP family is closely related to neutrophil division, proliferation, differentiation and maturation as well as granular protein formation (19, 20). These results were also verified in our study. The expression of CEBPA and CEBPE was significantly up-regulated in G2-G4 cells, and reached the peak in G3 subset (**Figure 7C**). Meanwhile, CEBPB and CEBPD were highly expressed in G5 cells (**Supplementary Figures 4A, B**). In addition, the genes (NFKB1, NFKB2, RELA, RELB, REL) of NF-κB pathway have higher regulation intensity in G5c cells (**Figure 7E** and **Supplementary Figures 4C, D**), confirming that they play an important role in regulating neutrophil apoptosis.

In addition, the intensity of CEBPA and CEBPE regulation was higher in UCB than in healthy adult neutrophils (**Figure 7D**), suggesting that neutrophils of UCB were more naïve and differentiative potential. Different from the expression of NFKB2, RELA and RELB transcription factors, the expression of NFKB1 and REL were enhanced in UCB (**Figure 7F**, **Supplementary Figures 4E, F**). Interestingly, the intensity of HIF1A regulation peaked in G4 cells (**Supplementary Figure 4G**). Moreover, HIF1A has strong regulation role in UCB than in adults (**Supplementary Figure 4H**).

Furthermore, ZNF276, ZNF319, and ZNF354A were detected (**Figure 7G**), whose regulatory role was unclear in the published literatures. These transcription factors exhibited significant differences in differentiation and maturation of neutrophils, and their functions required further research.

## Discussion

Fetal immune system development was a special process. Placental microenvironment and pregnancy hormones play a key role in shaping the phenotype, function, metabolism and transcription of neutrophils in UCB (21, 22). They are the reasons for the different genotypes of neutrophils in UCB compared to healthy adults. In the previous literatures, such differences were considered as functional defects or impaired functions of neutrophils in UCB (23, 24). However, we prefer to regard the differences as an adaptive physiological change during cell development.

Using scRNA-seq, we found that neutrophils of UCB showed commonality and characteristics compared to healthy adults. The classification of UCB neutrophils and healthy adult neutrophils was conserved, and the neutrophils were divided into six subpopulations with identical genetic markers. Different from the studies by Xie et al. (14)and Huang et al (16), we found the existence of G2 cell populations with proliferative functions and G3 and G4 subpopulations, in addition to mature G5a, G5b and G5c subsets in UCB and adults. This was consistent with reports that immature neutrophils account for a higher proportion in UCB (25). In particular, G2 and G3 subsets could predict diseases such as sepsis, infection, inflammation and tumor metastasis. In addition, compared with healthy adults, GO-BP analysis of neutrophil subgroups in UCB was abundant in cell activation, degranulation and secretion, and short of interferon-related pathways, immune response and cytokine response. This could explain why newborns were more susceptible to infection, as stated in many studies (24, 26, 27).

Moreover, we found significant differences in the function and quantity of the G5b subset of UCB compared to healthy adults. G5b subset could be rapidly corresponding and amplified facing infection-related diseases, which played a key role in the immune defense (14). We hypothesized that neutrophils in bone marrow were reprogrammed under the continuous stimulation of the external microbial environment after birth. These complex environmental factors, such as various bacterial components, inflammatory cytokines, toxins and so on, should be the key to the maturation of the immune system, and these factors might regulate the amplification of G5b in UCB and made the function close to adults. The weakness of G5b in UCB might partially explain the inhibited innate immune response and poor rapid mobilization ability of neutrophils of neonates in the face of infection.

Furthermore, we noted a special neutrophil subset of G5c2 (CD52^+^) in G5c cells, which was almost absent in UCB. CD52 was generally expressed on the surface of immune cells such as lymphocytes, eosinophils and natural killer cells, and is involved in graft-versus-host disease (28–30). Lin et al. demonstrated the neutrophils with low expression of CD52 complement mediated lysis and resulted in neutropenia in the presence of Alemtuzumab (31). Our study confirmed that the CD52 gene was specifically expressed in mature neutrophil G5c cells. Interestingly, the GO-BP and pathway analysis of G5c2 were more similar to that of G3 subgroup. We hypothesized that this subgroup might be directly differentiated from G3 cells by exposure to external microorganisms, and to be a stable subset of cells with memory function. Primitive neutrophils may have evolved into the G5c2 subgroup in response to exposure to microbe-rich environments. These speculations still required further validation.

Apart from that, we found that mitochondria dependent metabolisms were stronger in the early stages of neutrophil proliferation and differentiation (G2, G3, G4), and significantly in UCB. This suggested that mitochondrial metabolism might be more important than glycolysis metabolism in immature neutrophils different from previous studies (18, 32, 33). However, mature neutrophils were considered to be primarily glycolytic metabolites. The transformation of neutrophil metabolic phenotype still needs further exploration. Oxidative stress was a consequence of excessive oxidants or free radicals, which was mainly reactive oxygen species and reactive nitrogen (34). Excessive oxidative stress may lead to a variety of pregnancy-related disorders, most notably pre-eclampsia and intrauterine growth restriction (35, 36). Mature neutrophils in UCB were significantly down-regulated in ROS production and related pathways such as NAPDH, and respiratory electron transport chain. This might be related with the variety of antioxidants secreted by the placenta (21), which transmitted directly or indirectly to the fetus to prevent the effects of excessive oxidative stress on the fetus.

In the neutrophil transcription factor regulatory network, C/EBP family, NF-κB transcription was expressed differently between UCB and adults. CEBPA played an important role in regulating neutrophil lineage and early differentiation, and CEBPE was also involved in the formation of specific particles and gelatinase particles (19, 20). The upregulation of CEBPA and CEBPE in UCB suggested that neutrophils in UCB were naive and have more differentiational potential. The NF-κB transcription factor family regulated a variety of biological processes, including many aspects of immune function (37–39). NFKB1 and REL played an important role in B lymphocytes and regulation of T cell proliferation (40, 41). The up-regulation of NFKB1 and REL in UCB may indicate that these two transcription factors might be involved in regulating the early proliferation and differentiation of neutrophils. Besides, it was well known that bone marrow hematopoietic stem cells were maintained in a hypoxic environment, and hypoxic transcription factors were highly expressed in order to adapt to the hypoxic environment. In our study, HIF1A was gradually upregulated and reached its peak at G4 cells instead of G2 cells, suggesting that HIF1A might play an important regulatory role in the neutrophilic maturation, more than be adapt to environmental hypoxia.

We applied scRNA-seq for the first time to study cord blood neutrophils to provide a comprehensive transcriptome view of them. Neutrophils contributed to the first line of defense against infection. Therefore, accurate functional evaluation of neutrophils in healthy neonates could provide theoretical basis for early evaluation and early warning of infection-related diseases in neonates. Neutrophils allotransplantation could treat neutropenia and dysfunction related diseases (such as malignant tumors, autoimmune diseases, etc.) (42, 43). Compared with adults, neutrophils from UCB were more suitable for allogeneic cell transplantation due to their convenient selection, more primitive, less differentiation potential and less graft-versus-host reaction. Our previous study found that cord blood neutrophils were a complex population with different functional subsets. However, single-subgroup allotransplantation was theoretically more controllable than multi-subgroup allotransplantation. In addition, studies have shown that different subgroups of neutrophils have the potential to convert to each other under different stimuli (14). Therefore, in future studies, we could treat different types of diseases through precise and targeted training (such as cytokines, toxins and bacterial bones et al) of cord blood neutrophils with the characteristic of high differentiation potential. In conclusion, a comprehensive analysis of the heterogeneity and functional diversity of cord blood neutrophils has been carried out, providing potential opportunities for early diagnosis and treatment in subsequent diseases.

## Method

### Ethical approval

This study was approved by the Medical Ethics Committee of Suzhou Hospital affiliated to Nanjing Medical University. UCB and healthy adult volunteers provided consent for all experiments that involved human blood. We collected blood samples from UCB and cubital vein of healthy donors. Experiments were conducted according to approved guidelines.

### Extraction of umbilical UCB and adult peripheral blood neutrophils

Peripheral blood of healthy adults was obtained from cubital vein of volunteer. UCB was taken from the umbilical cord arteries after delivery and ligated during the third stage of labor before delivery of the placenta. 4ml of blood sample was stored in heparin anticoagulant tubes and separated within 2 hours. Negative selection for neutrophil was performed using a magnetic bead separation kit according to the specification (Stemcell, Vancouver, Canada). The neutrophils extracted with magnetic beads were washed twice in PBS, and then centrifuged to discard the supernatant. The extracted neutrophils were cultured in RPMI 1640 (Gbico, Canada) containing 10% fetal bovine serum (Gbico, New Zealand).

### Single-cell RNA sequencing

Single-cell transcriptome information was captured (from 10 sample sources) using the BD Rhapsody system. Through a limited dilution method, the single-cell suspension was randomly assigned to 200,000 micropores. In order to pair cells in micropores with the beads containing oligonucleotide barcodes, the beads were added to the saturated state. Micropores in the cells were used to hybridize mRNA molecules and the bar codes on the beads were used to capture oligonucleotides. After reverse transcription, ExoI digestion was performed in a test tube. An UMI was bound to each cDNA molecule at the 5’ end during cDNA synthesis to identify the source of the DNA. For full transcriptome libraries, BD Rhapsody uses random primers and extensions (RPE), amplification PCR (RPE), and WTA index PCR (WTA). The library was quantified using an Agilent high-sensitivity DNA chip on the Bioanalyzer 2200 and a Thermo Fisher Scientific qubit high sensitivity DNA analysis. The sequencing was done on a 150-bp paired-end run by an Illumina sequencer (Illumina, San Diego, CA).

### Single-cell RNA statistical analysis

To achieve high-quality data, we filter adapter reads and delete low-quality reads by using the default parameters and FastP. With the application UMI-tools, the single-cell transcriptome recognizes the whitelist of cell barcodes. Data were mapped to the human genome (Ensemble version 91) using STAR mapping. To obtain the UMI counts for each sample, the UMI-tools standard pipeline was used. Cells with more than 200 expressed genes and mitochondrial UMI rate below 10% passed cell quality filtering and mitochondrial gene analysis. We used the Seurat package (version: 3.1.4, https://satijalab.org/seurat/) to normalization and regression of the cell, after scaling the data. The criteria for PCA construction was the first 2000 highly mutated genes, and tSNE and UMAP construction was based on the first 10 PCCS. We used Harmony to eliminate potential batch effects, since samples are batch processed and sorted. Based on the graph clustering method, we obtained the clustering results of unsupervised cells based on the top 10 subjects. Marker genes for the result were calculated using Findallmarker function and Wilcox rank-sum test algorithm (lnFC> 0.25; Pvalue < 0.05; Min. PCT > 0.1).

### GO-pathway enrichment analysis

In this experiment, GO analysis was conducted to clarify the biological significance of unique genes in the significant or representative profiles of the differentially expressed genes (44).The GO annotations were downloaded from NCBI (http://www.ncbi.nlm.nih.gov/). We used Fisher’s exact test to identify significant GO categories and FDR to correct the p-values.

By analyzing KEGG’s pathway database, we determined the significant pathways of the differential genes. Fisher’s exact test is used to select the significant pathway, and the P-value and FDR are used to define the threshold of significance (45).

### SCENIC analysis

Three new R packages are used in SCENIC. The first is GENIE3, which identifies TF targets based on co-expression. The second is RcisTarget, used to perform motif enrichment. The third and final option is AUCell, a method of assessing regulon activity within single cells. We evaluate transcription factor regulatory strength using the 20-thousand motif database of RcisTarget and GRNboost and We also use single-cell regulatory networks (pySCENIC, v0.9.5) and clustering workflows (pySCENIC). Codes and tutorials for CENIC are available at http://scenic.aertslab.org.

### Co-regulated gene analysis(scGeneModule)

We used the find gene modules function of monocle3 with the default parameters for discovering gene co-regulation networks.

### Pseudo-time analysis

Using BEAM analysis, we examined the genes that control how branches grow based on a quasi-temporal analysis. We screened the expression count of primary cells before Monocle analysis, and selected the marker genes of Seurat (version 3.1.4) for clustering results. We used BEAM analysis to analyze the genes that determine the fate of branches on the basis of quasi-temporal analysis.

### Function score

We scored individual neutrophils for genetic characteristics of certain biological functions. The cells’ corresponding function scores were scored using the mean normalized expression of the corresponding genes.

### Identification of DEGs

To identify DEGs, we used the FindMarkers function (test). Use = “bimod”, logfc.threshold=log[1.5], min.pct = 0.01). The analysis of gene ontology was carried out using the R package top GO. Disease analysis was conducted by using Fisher’s Exact Test based on DisGeNET (http://www.disgenet.org).

### Chemotaxis of neutrophils

In a 35 mm petri dish, 2.7mL agarose (1.2% agarose, 50% HBSS containing H2CO3, 50% RPMI1640 containing heat-inactivated FBS) was added, and stood at room temperature for 5min. Then place the petri dish in a 4° refrigerator for 30-60min. Before adding samples, three holes with a diameter of 3.5mm and a spacing of 2.8mm were drilled on the obtained gel using a hole punch. Chemotactic peptide (fMLP, 10μ L) was added to the middle hole, 10ul cell suspension (10^7 cells/mL) was added to both sides, and incubated for 2h at 37° in an incubator containing 5% CO2. At the end of culture, the chemotactic distance was measured and observed under an optical microscope.

### Flow cytometry

Neutrophils were resuspended in precooled PBS at a cell concentration of 5x10^6 cells/mL (100 μL PBS, 5x10^5 cells/tube). Antibodies are used according to the manufacturer’s agreement. After incubation for 0.5h, FACS Canto II cell analyzer (BD Biosciences) was used to detect neutrophils (BD Biosciences). FlowJo software was used for data analysis. Respiratory oxygen explosion of neutrophils was detected by CM-H2DCFDA (Solarbio, China). The plasma membrane expression of CD35(BD, USA) and CD63(BD, USA) was measured to determine degranulation of secretory granules. Neutrophil phagocytic ability was detected by Phagocytosis kit (Red Zymosan) (abcam, USA) and neutrophils were incubated with the media (1640, 10%.FBS) for 2h. Analyzing apoptosis requires annexin V and 7-AAD (BD, USA), as per manufacturer’s protocol.

### Statistical analysis

As a general rule, experiments are compared using a two-tailed, unpaired T-test. In each graph, the values represent the mean plus standard deviation, and P<0.05 was considered statistically significant. All experiments were repeated at least three times. The statistical analysis and graphics were completed with GraphPad Prism (GraphPad) and R (Statistical computation of R items).

## Data availability statement

The data presented in the study are deposited in the NCBI repository, accession number PRJNA864027. The SRA records are accessible with the following link: https://www.ncbi.nlm.nih.gov/sra/PRJNA864027

## Ethics statement

This study was reviewed and approved by Suzhou Hospital affiliated to Nanjing Medical University. Written informed consent to participate in this study was provided by the participants’ legal guardian/next of kin.

## Author contributions

BS and YC designed the study and wrote paper. YC, JH, ZG, ZZ, YS, LuL, YuY, YaY and LuL performed the RNA-seq data analysis. JH and ZG performed experiments. LuL and YaY performed collection of clinical data. YC and BS performed the statistical analysis. All authors contributed to the article and approved the submitted version.

## Funding

This study was supported by the National Natural Science Foundation of China, No.82072217, 81772135 and U21A20370; by the Jiangsu Natural Science Foundation, BK20201178.

## Conflict of interest

The authors declare that the research was conducted in the absence of any commercial or financial relationships that could be construed as a potential conflict of interest.

## Publisher’s note

All claims expressed in this article are solely those of the authors and do not necessarily represent those of their affiliated organizations, or those of the publisher, the editors and the reviewers. Any product that may be evaluated in this article, or claim that may be made by its manufacturer, is not guaranteed or endorsed by the publisher.
