# Supplementary material for: Primitive genotypic characteristics in umbilical cord neutrophils identified by single-cell transcriptome profiling and functional prediction
Source: Front Immunol. 2022 Aug 29;13:970909. doi: 10.3389/fimmu.2022.970909 (PMC9464600; doi:10.3389/fimmu.2022.970909)
Supplement: Supplementary file 1 [file DataSheet_1.docx]

**Supplementary Information for**

**Primitive genotypic characteristics in umbilical cord neutrophils identified by single-cell transcriptome profiling and functional prediction**

Yi Chen, Jiamin Huang, Zaiwen Guo, Zhechen Zhu, Yiming Shao, Linbin Li, Yunxi Yang, Yanzhen Yu, Lu Liu, Bingwei Sun

*** Corresponding author**

Email: sunbinwe@hotmail.com

**This file includes:**

**Supplementary Figure1 to 4**


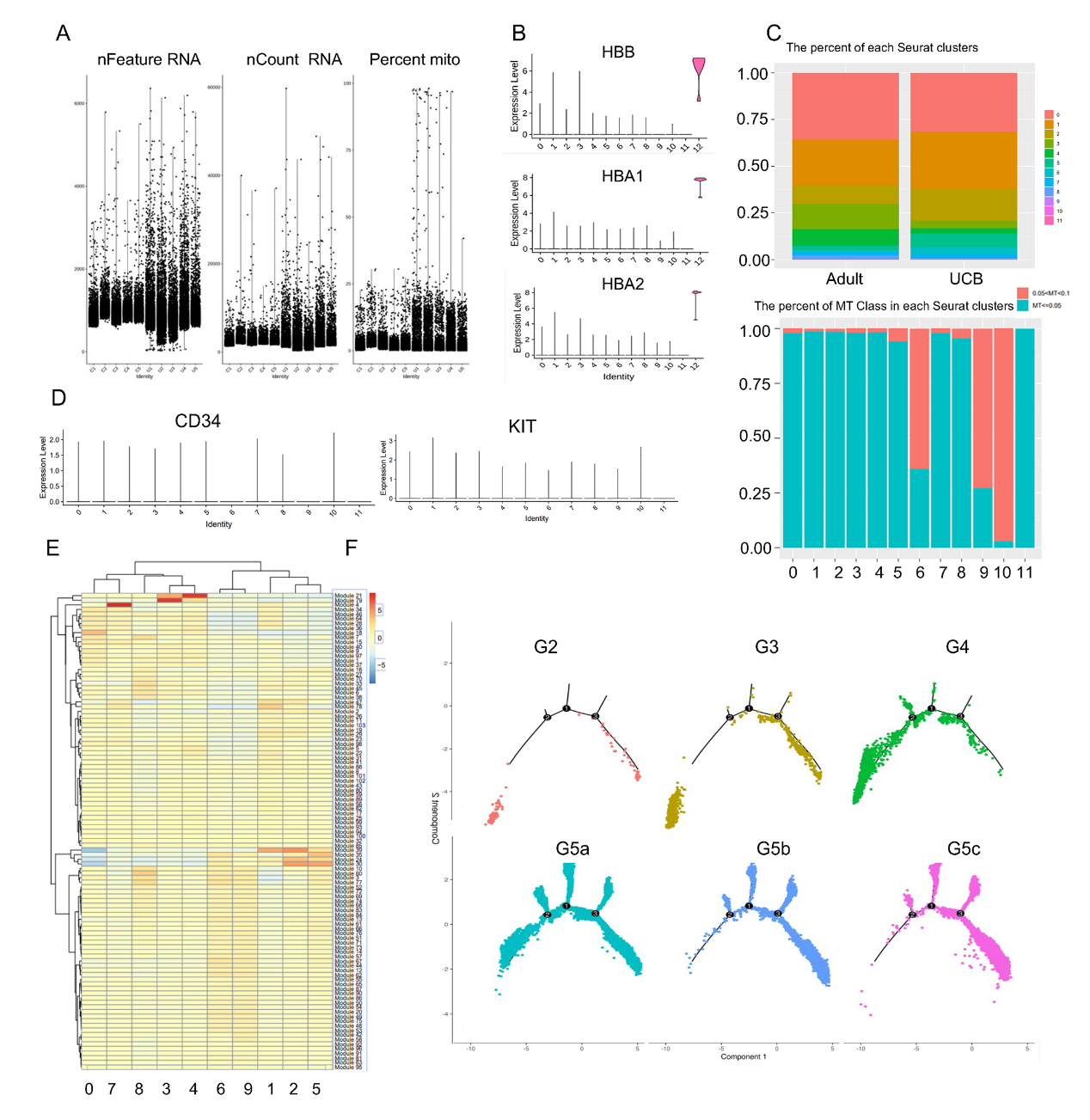
**Supplementary Figure 1: The quality control of neutrophils and the characteristics of developmental differentiation under homeostasis based on scRNA-seq.**

1. An overview of the number of genes, the number of UMIs, and the percentage of mitochondria in each sample in all samples.
2. The proportion information of subsets (the picture above) and mitochondrial genes (the picture below) in all samples.
3. Violin diagrams of genes expression CD34 and KIT in subsets.
4. Analysis for subsets based on single cell gene module.
5. Reference diagram of development direction for pseudo-time analysis.
6. Diagram illustrating development direction of pseudo-time analysis of individual cells in each subset.


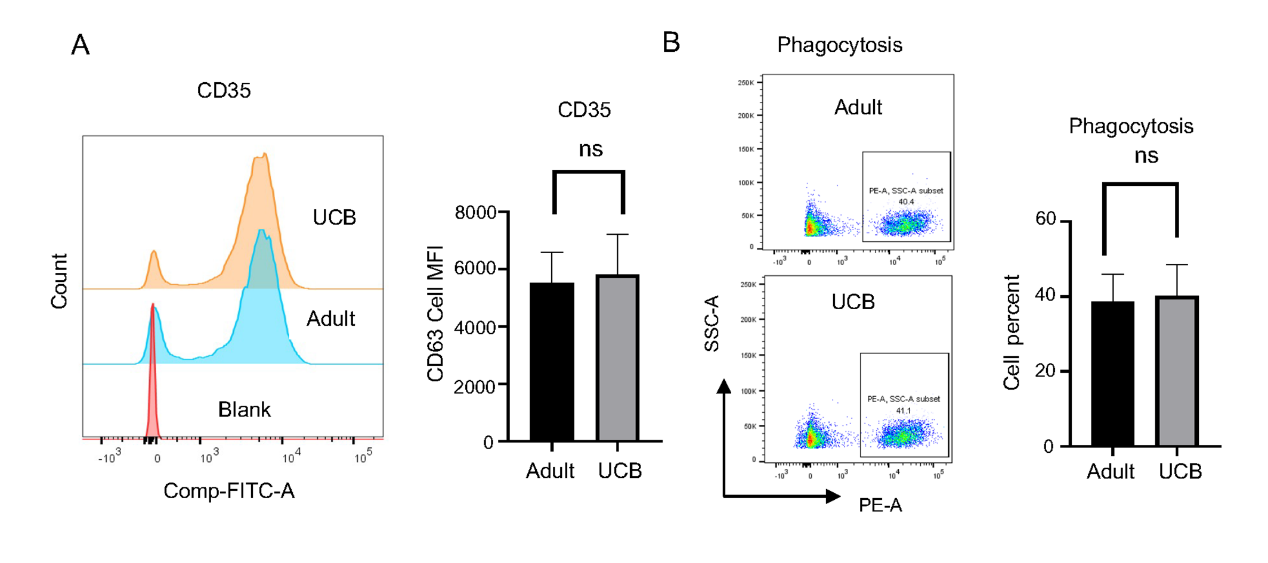
 **Supplementary Figure 2: Functional experiments of neutrophils between UCB and adult in vitro.**

1. The expression levels of CD35 in neutrophils of healthy adult and UCB.
2. Neutrophil phagocytosis in healthy adults and UCB.

Data represent means ± s.d. (n= 3–5) of two independent experiments. *p < 0.05, **p < 0.01, ***p < 0.001, ns, not statistically.


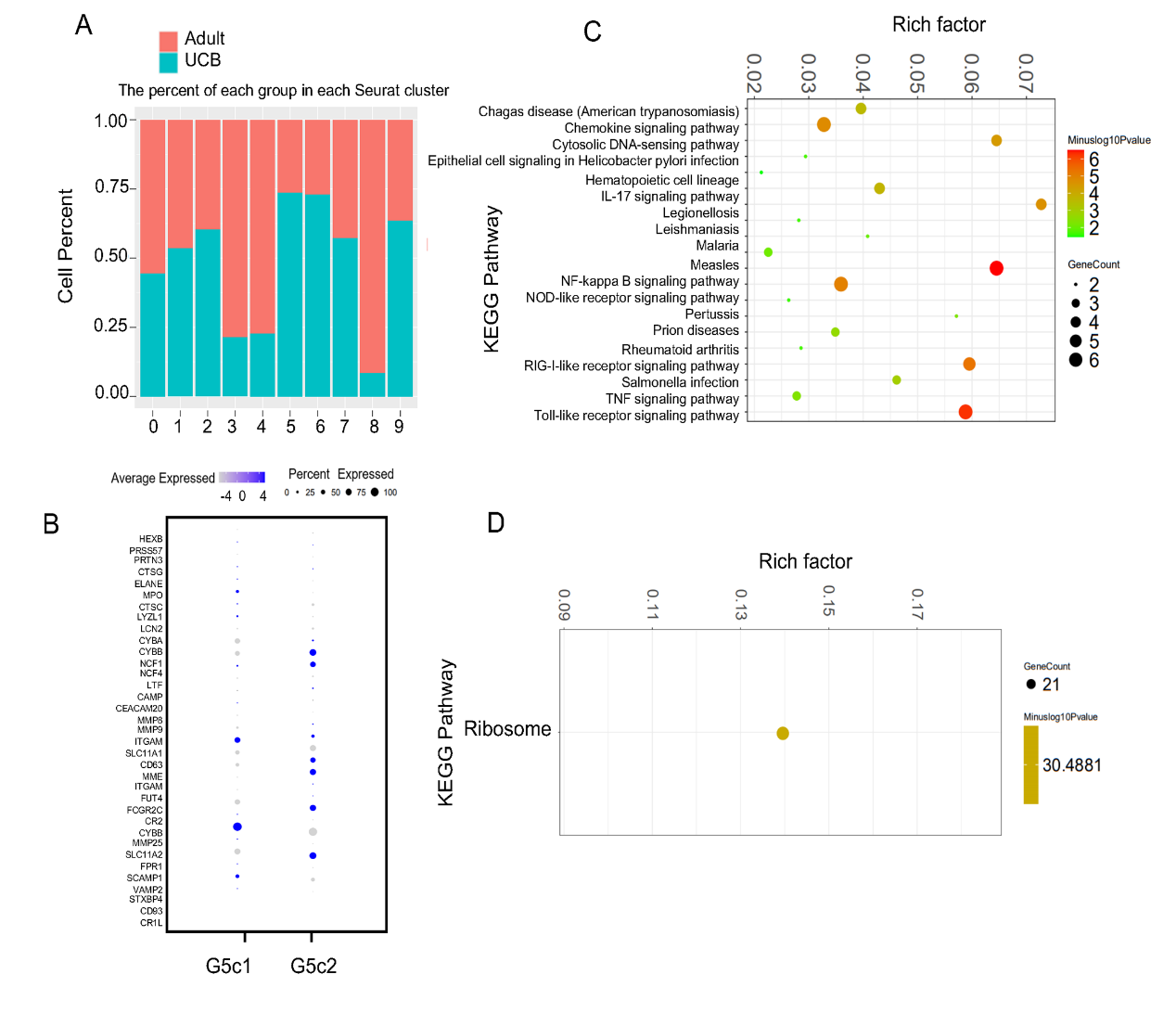


**Supplementary Figure 3: G5c subgroup and their functional characteristics.**

(A) The proportion information of neutrophils between healthy adult and UCB in each subset. 0,7 and 8 clusters were considered as G5c, and 8 cluster was redefined as G5c2.

(B) Scaling diagram of different expressions of neutrophil particle genes in G5c subgroups.

(C-D) Pathway analysis of G5C. (C) Enrichment pathways based on G5c1 differential gene(up).

(D) Enrichment pathways based on G5c2 differential gene(up).


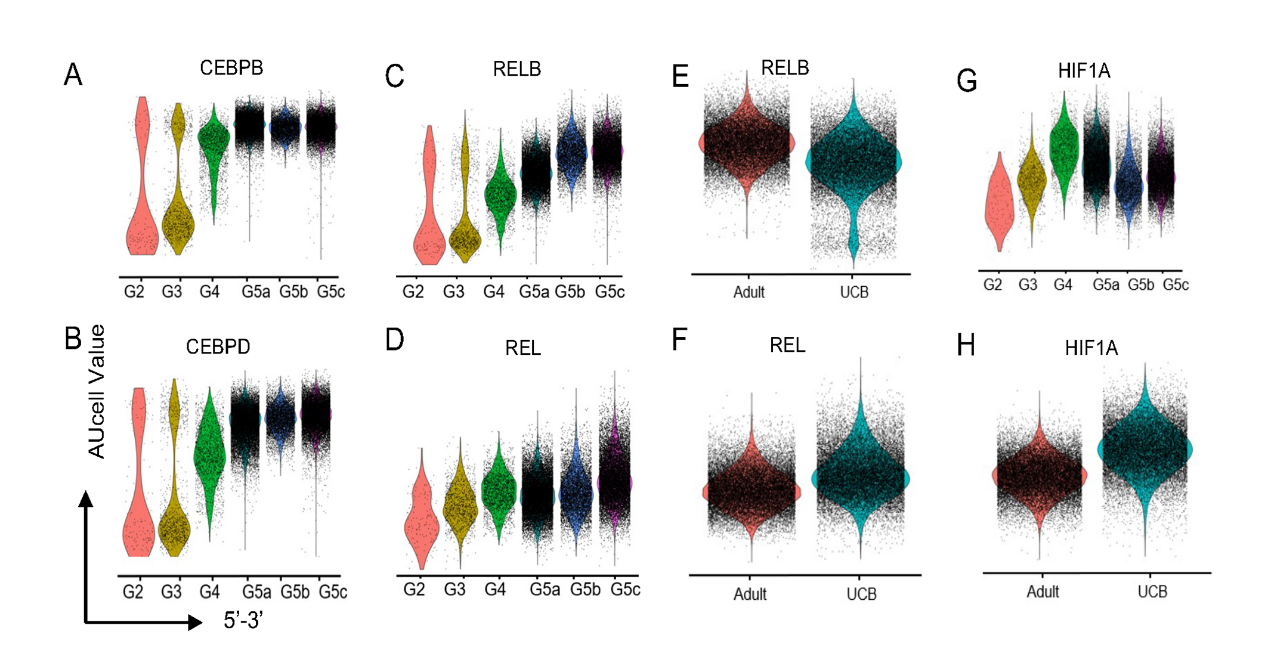
 **Supplementary Figure 4:** **Characteristics of transcription and communication in neutrophil subsets.**

**(A-H)** Violin plots of different activity of transcription factors in neutrophil subsets and different groups.
